# Supplementary material for: HIVprotI: an integrated web based platform for prediction and design of HIV proteins inhibitors
Source: J Cheminform. 2018 Mar 9;10:12. doi: 10.1186/s13321-018-0266-y (PMC5845081; doi:10.1186/s13321-018-0266-y)
Supplement: Supplementary file 2 — Additional file 2. Source code of HIVProtI web server. [file 13321_2018_266_MOESM2_ESM.zip › HIVprotI_Source-code/hivprotI/weka-3-6-6/doc/weka/datagenerators/classifiers/regression/package-summary.html]

weka.datagenerators.classifiers.regression


---


|  |  |  |
| --- | --- | --- |
| **Overview** \| **Package** \| Class \| **Tree** \| **Deprecated** \| **Index** \| **Help** \| **Weka's home** | |  |
| **PREV PACKAGE**   **NEXT PACKAGE** | **FRAMES**    **NO FRAMES**     **All Classes** |  |


---

## Package weka.datagenerators.classifiers.regression

| **Class Summary** | |
| --- | --- |
| **Expression** | A data generator for generating y according to a given expression out of randomly generated x.  E.g., the mexican hat can be generated like this:  sin(abs(a1)) / abs(a1)  In addition to this function, the amplitude can be changed and gaussian noise can be added. |
| **MexicanHat** | A data generator for the simple 'Mexian Hat' function:  y = sin\|x\| / \|x\|  In addition to this simple function, the amplitude can be changed and gaussian noise can be added. |

---


|  |  |  |
| --- | --- | --- |
| **Overview** \| **Package** \| Class \| **Tree** \| **Deprecated** \| **Index** \| **Help** \| **Weka's home** | |  |
| **PREV PACKAGE**   **NEXT PACKAGE** | **FRAMES**    **NO FRAMES**     **All Classes** |  |


---
